# Lipid-modifying pharmacotherapy in diabetes mellitus: A protocol for a systematic review and network meta-analysis of randomised trials

**DOI:** 10.1371/journal.pone.0354259

**Published:** 2026-07-23

**Authors:** Xudong Zhao, Cheng Tang, Tianqi Yuan, Jiafan Chen, Cuijuan Shi, Xiaoyu Liu, Ruigeng Yang, Yushang Zhi, Wenrui Huang, Chunyan Zhu, Yu Chen, Shidong Wang

**Affiliations:** 1 Department of Endocrinology and Nephrology II, Dongzhimen Hospital, Beijing University of Chinese Medicine, Beijing, China; 2 Qihuang College of Beijing University of Chinese Medicine, Beijing, China; 3 Department of Cardiology, Guang’ anmen Hospital, China Academy of Chinese Medical Sciences, Beijing, China; 4 Department of Endocrinology and Metabolism, First Teaching Hospital of Tianjin University of Traditional Chinese Medicine, Tianjin, China; 5 National Clinical Research Center for Chinese Medicine Acupuncture and Moxibustion, Tianjin, China; 6 Graduate School of Beijing University of Chinese Medicine, Beijing, China; 7 Shenzhen Traditional Chinese Medicine Hospital, Guangzhou University of Chinese Medicine, Shenzhen, China; 8 Peripheral Vascular Department, Dongzhimen Hospital, Beijing University of Chinese Medicine, Beijing, China; Università degli Studi di Milano, ITALY

## Abstract

**Introduction:**

The global prevalence of diabetes mellitus (DM) has risen markedly, with patients facing a substantially higher risk of atherosclerotic cardiovascular disease (ASCVD) and related mortality. Although lipid-modifying agents, including statins, fibrates, cholesterol absorption inhibitors, proprotein convertase subtilisin/kexin type 9 inhibitors (PCSK9i), bromodomain and extra-terminal motif inhibitors (BETi), and adenosine triphosphate-citrate lyase inhibitors (ACLi) continue to emerge, their comparative efficacy and safety in patients with DM remain uncertain. This study aims to address this gap through a comprehensive network meta-analysis (NMA) of randomized controlled trials (RCTs) evaluating cardiovascular outcomes, lipid-modifying efficacy, and safety among these therapies.

**Methods and analysis:**

A systematic search will be performed in PubMed, Web of Science, Scopus, the Cochrane Central Register of Controlled Trials (CENTRAL), Embase (via Ovid), International Clinical Trials Registry Platform (ICTRP) and ClinicalTrials.gov from inception, without language or regional restrictions. Eligible studies will include parallel-design RCTs comparing monotherapy or combination therapy. The primary outcome will be cardiovascular events (composite and individual), and secondary outcomes will include cardiac biomarkers, ASCVD risk scores, lipid parameters (TC, LDL-C, HDL-C, TG) and safety outcomes. Two reviewers will independently conduct study selection, data extraction, and quality assessment using the Cochrane Risk of Bias 2 (RoB 2.0) tool, with evidence certainty evaluated via the Confidence in Network Meta-Analysis (CINeMA) framework. Data will be standardized where appropriate, and analyses, including assessment of publication bias, will be conducted using Stata 18 and R. The protocol is registered with PROSPERO, and findings will be disseminated through peer-reviewed publication.

**Registration:** PROSPERO CRD420251163974.

## 1. Introduction

Diabetes mellitus (DM) represents a major global health challenge, currently affecting more than half a billion individuals worldwide—a number that continues to grow, largely driven by lifestyle changes [1]. As of 2024, an estimated 588.7 million people were living with DM [2]. The increasing prevalence, coupled with the lifelong need for pharmacological management, places a substantial burden on healthcare systems. As the disease progresses, patients frequently develop a spectrum of complications and comorbidities [3,4]. Moreover, individuals with diabetes are at a significantly elevated risk of atherosclerotic cardiovascular disease (ASCVD) and related adverse outcomes, which substantially contribute to overall mortality [5]. Consequently, therapeutic strategies should not only aim to improve metabolic parameters such as lipid profiles but also focus on reducing cardiovascular risk while minimizing potential adverse drug effects that may constrain treatment options.

Lipid management constitutes a cornerstone of diabetes care. DM is commonly associated with characteristic disturbances in lipid and apolipoprotein metabolism [6]. Low-density lipoprotein cholesterol (LDL-C) is recognized as a principal modifiable risk factor and the primary therapeutic target for ASCVD prevention [7–9]. Recent clinical guidelines have further underscored the relevance of additional lipid parameters in cardiovascular risk assessment, including triglycerides (TG), high-density lipoprotein cholesterol (HDL-C), and apolipoprotein B (ApoB)-containing lipoproteins [10–14]. Owing to their role in cholesterol transport and interaction with the vascular endothelium, these lipids are involved in multiple atherogenic processes that ultimately lead to lipid accumulation and the formation of atherosclerotic plaques [15,16].

Lipid-lowering therapies have been shown to reduce the risk of adverse cardiovascular outcomes among patients with established cardiovascular disease [17]. Currently, a broad spectrum of lipid-modifying agents is available, encompassing traditional therapies such as statins and fibrates, as well as more recently developed agents, including proprotein convertase subtilisin/kexin type 9 inhibitors (PCSK9i) and adenosine triphosphate-citrate lyase inhibitors (ACLi) [18–21]. Nevertheless, head-to-head RCTs directly comparing these agents in individuals with DM remain scarce. Consequently, lipid management in diabetic populations continues to rely largely on evidence extrapolated from studies conducted in the general dyslipidaemic population and from multiple independent RCTs [6].

Previous meta-analyses have examined the effects of specific drug classes, such as statins [22], fibrates [23], n-3 fatty acids [24,25], TG-lowering agents [21], and PCSK9 inhibitors [26]. However, these investigations were generally confined to class-level assessments, capturing only the aggregated effects of agents with shared mechanisms of action compared with control groups. As such, they offer limited clinical guidance for selecting specific agents within or across drug classes. Furthermore, the inclusion of non-placebo comparators in some studies [22] complicates the interpretation and synthesis of the available evidence. Given the expanding therapeutic landscape, both clinicians and patients are often confronted with multiple lipid-lowering options, underscoring the need for an integrated and comparative assessment of these therapies.

Accordingly, this systematic review and network meta-analysis (NMA) is designed to provide a comprehensive comparison of the efficacy and safety profiles of all currently available lipid-modifying agents in patients with diabetes. By jointly analyzing direct and indirect evidence, this study aims to generate an overarching comparative evaluation of these therapies on major adverse cardiovascular events (MACE), individual cardiovascular outcomes, lipid parameters, and safety outcomes in diabetic populations, thereby supporting evidence-based clinical decision-making and informing future research directions.

## 2. Methods and analysis

This study will be conducted as a systematic review and NMA. The protocol has been developed in accordance with the Preferred Reporting Items for Systematic Review and Meta-Analysis Protocols (PRISMA-P) guidelines [27–29] and has been prospectively registered in PROSPERO (CRD420251163974). The review is currently at the protocol stage and has not yet commenced beyond protocol development. The final review will adhere to the PRISMA 2020 reporting standards and the PRISMA extension for network meta-analyses (PRISMA-NMA) [30,31]. Any major amendments to this protocol will be transparently documented and reported in the final publication.

### 2.1. Inclusion criteria

Only RCTs with a parallel-group design will be included, in which participants are randomly assigned to receive either monotherapy or combination lipid-modifying therapy. Trials comparing lipid-modifying agents with placebo as an add-on treatment, when administered alongside a non-randomized pharmacological or lifestyle intervention, will be considered as head-to-head comparisons between the active agent and placebo. Studies employing comparators consisting solely of standard care or no treatment will be excluded. Reporting of cardiovascular outcomes with explicitly defined criteria will be mandatory. For primary cardiovascular event outcomes, only RCTs with a minimum follow-up duration of ≥12 months will be included, to ensure sufficient capture of ASCVD events. For secondary lipid efficacy outcomes, randomized controlled trials with a minimum follow-up duration of ≥6 months will be included.

Eligible studies will include peer-reviewed publications, encompassing both primary trial results and pre-specified secondary analyses. In cases of duplicate reporting, the most comprehensive or latest version will be included. No language or regional restrictions will be applied. Participants of all ages, sexes, ethnicities, and geographic locations will be eligible, whereas studies enrolling individuals with severe comorbidities (e.g., active malignancy or dialysis dependence) will be excluded.

Interventions of interest will comprise drugs primarily indicated for lipid modification, including those approved, under regulatory review, or in clinical development, according to agencies such as the U.S. Food and Drug Administration (FDA) and the European Medicines Agency (EMA). Eligible drug classes will include HMG-CoA reductase inhibitors (statins), cholesterol absorption inhibitors, PCSK9i, fibrates, nicotinic acid, omega-3 fatty acids, bile acid sequestrants, ACLi, inclisiran, bromodomain and extra-terminal motif inhibitors (BETi, in clinical development), and other relevant lipid-modifying agents identified during the literature search. The development status of non-marketed drugs (i.e., under regulatory review or in clinical development) will also be noted in the final results.

Participants receiving antidiabetic or antihypertensive therapies as background treatments will be eligible, provided they are balanced across intervention arms. Acceptable concomitant medications will include biguanides, insulin, sulfonylureas, thiazolidinediones, α-glucosidase inhibitors, dipeptidyl peptidase-4 inhibitors (DPP-4i), glucagon-like peptide-1 receptor agonists (GLP-1 RAs), sodium-glucose cotransporter-2 inhibitors (SGLT2i), angiotensin-converting enzyme inhibitors (ACEIs), angiotensin II receptor blockers (ARBs), calcium channel blockers (CCBs), β-blockers, α-blockers, diuretics, and other relevant concomitant therapies identified through the systematic search.

### 2.2. Exclusion criteria

If a study meets any of the following conditions, it will be excluded: the control group received non-pharmacological intervention or received no treatment at all; the study employed crossover trials or non-inferiority designs; the intervention measures involved drugs that have been withdrawn from the market; the full text is unavailable, or only the abstract of the conference is available.

### 2.3. Search strategy

We will conduct a comprehensive systematic search across PubMed, Web of Science, Scopus, the Cochrane Central Register of Controlled Trials (CENTRAL), Embase (via Ovid), International Clinical Trials Registry Platform (ICTRP) and ClinicalTrials.gov to identify relevant RCTs investigating lipid-modifying agents in patients with diabetes. The search will cover all records from database inception to the date of the final search. A sample PubMed search strategy is provided in Table 1 in S1 Text.

All relevant keywords and medical subject headings (MeSH) will be combined using Boolean logical operators. Synonyms and related terms will be linked with the *OR* operator, while an asterisk (*) will be applied as a truncation symbol to capture variations of a root word.

In addition, a comprehensive search of the relevant meta-analyses will be performed to identify potentially pertinent articles in the reference lists (Table 2 in S1 Text). Abstracts presented at major scientific meetings—specifically those of the European Association for the Study of Diabetes (EASD) and the American Diabetes Association (ADA)—will also be reviewed for information on clinical trials. Moreover, manual searches will be performed on regulatory agency websites, including those of FDA and EMA, to identify registered but unpublished studies.

The search strategy will be independently developed by two reviewers (XZ and YZ) and subsequently reviewed by additional team members (CT and CS) for completeness and accuracy. Following independent term formulation, a consensus meeting will be held in which both reviewers present and discuss their proposed strategies. When agreement has been reached, the unified strategy will be adopted. In cases of disagreement, the issue will be resolved by an adjudication panel (CS, XL, and RY), serving as impartial arbiters to ensure objectivity and methodological rigor. Finally, the comprehensive literature search will be independently executed by two reviewers (XZ and JC).

### 2.4. Study selection

**Fig 1:**
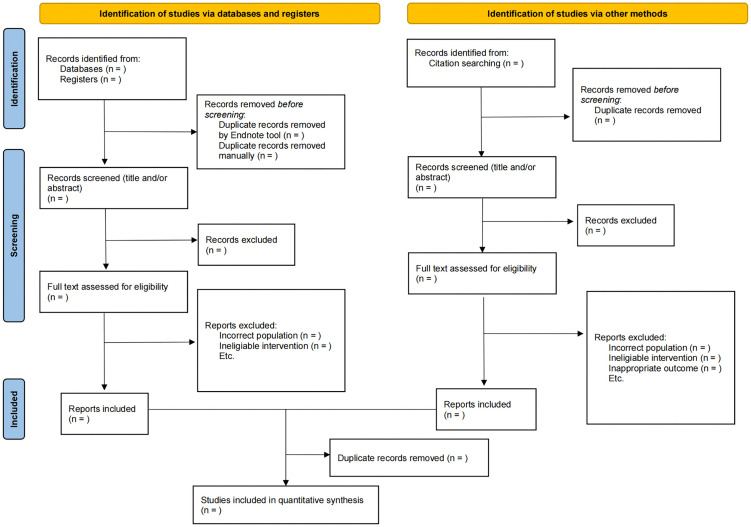
An overview of the research selection process (PRISMA).

All references retrieved from the databases will be managed using EndNote 20 (Clarivate Analytics, Philadelphia, PA, USA). Both studies identified through database searches and those obtained from citations of relevant network meta-analyses will be imported into the reference library. After the removal of duplicates, two reviewers (XZ and JC) will independently screen the titles and then abstracts of all records. Discrepancies between reviewers will be resolved through discussion, and any unresolved disagreements will be adjudicated by a third reviewer (SW). The full texts of potentially eligible studies will then be retrieved and evaluated against the predefined inclusion and exclusion criteria using EndNote 20. The overall process of study identification, screening, and selection will be documented and presented according to the PRISMA flow diagram (Fig 1) [30].

### 2.5. Data extraction

For each eligible study, data will be extracted using a predesigned standardized form. The following information will be collected: trial characteristics (first author, year of publication, country, trial registration number, and study design); participant characteristics (age, sex, sample size, duration of diabetes, body mass index, and HbA1c); intervention details (drug class, specific agent, dose, route of administration, and background therapy); cardiovascular events (e.g., MACE, total stroke), cardiac biomarkers (e.g., high-sensitivity cardiac troponin T/I [hs-cTnT/I], B-type natriuretic peptide [BNP], and N-terminal pro–B-type natriuretic peptide [NT-proBNP]), and ASCVD risk scores (e.g., the ASCVD Risk Estimator Plus developed by the American College of Cardiology) [32,33]; baseline and post-treatment lipid parameters, including total cholesterol (TC), LDL-C, HDL-C, and triglycerides (TG); and safety outcomes, including adverse events such as liver enzyme elevation, creatine phosphokinase elevation, and other reported events. For LDL-C, we will systematically record the quantification method of each included study, including direct laboratory measurement, or calculated via the Friedewald [34], Martin-Hopkins [35], or Sampson-NIH equation [36].

For studies reporting outcomes at multiple follow-up time points, outcome data from the longest available follow-up period will be extracted as the primary data source for cardiovascular outcomes analysis. For secondary outcomes and safety outcomes, data from all reported time points will be extracted, then data from the longest comparable time points will be statistically synthesized. Continuous variables will be presented as mean values, standard deviations (SD), where applicable, whereas categorical variables will be expressed as frequencies or percentages (%). We will evaluate series publication, assess all available data simultaneously, maximizing the extraction of data for a bias assessment precisely. For studies that reported results only in graphical form, numerical data will be extracted using WebPlotDigitizer (version 5.2; https://automeris.io/WebPlotDigitizer/). To ensure the reliability of the data extraction process, the data extraction form will be pilot tested by two independent reviewers (YZ and XL). Data extraction will be independently performed by two reviewers (XZ and CT), and discrepancies will be checked and resolved by a third reviewer (SW). In addition, if necessary, inquiries will be directed to the authors if there are any missing, unclear or insufficient data in the manuscript; otherwise, the study will not be considered. We will tabulate and narratively describe any relevant data that are deemed inappropriate to be quantitatively synthesised.

### 2.6. Outcome definition

The primary outcome of this study will be cardiovascular events, including both composite and individual endpoints (e.g., MACE and total stroke).

Secondary outcomes will include cardiac biomarkers (e.g., hs-cTnT/I, BNP, NT-proBNP); ASCVD risk scores; lipid parameters (TC, LDL-C, HDL-C, TG), and safety outcomes, including adverse events such as liver enzyme elevation, creatine phosphokinase elevation, and other reported events.

### 2.7. Quality assessment

The Cochrane Risk of Bias tool for randomized trials (ROB 2.0) will be used to evaluate the methodological quality of the included studies, covering five domains: randomization process, deviations from intended interventions, missing outcome data, measurement of the outcome, and selection of the reported result [37]. Trials will be judged to have an overall low risk of bias if all domains are rated as low risk, a high risk of bias if at least one domain is rated as high risk, and some concerns if neither condition is met. Two reviewers (XZ and CT) will independently assess the risk of bias, and disagreements will be resolved by consensus. The risk of bias assessments will be presented using a risk of bias summary bar graph.

### 2.8. Data synthesis

The data synthesis will primarily employ quantitative methodologies, supplemented by qualitative approaches when contextual relevance demands. To ensure comparability across studies, lipid parameters and cardiac biomarkers reported in different units will be standardized using internationally accepted conversion factors prior to analysis. Where applicable, results will be presented in both conventional units (e.g., mg/dL) and SI units (e.g., mmol/L) to improve interpretability for international readers. For continuous variables reported as mean ± SD, data will be directly extracted. When outcomes are presented as median (interquartile range [IQR]) or median (range), the underlying distribution will be evaluated based on the type of variable, the relative magnitude of the IQR compared with the mean, and whether nonparametric tests were employed in the original study. If the data appear to approximate a normal distribution (e.g., continuous physiological measures in large samples), means and SDs will be estimated using the formulas proposed by Wan et al. (2014) [38] and Luo et al. (2018) [39].

For continuous outcomes, results will be reported as standardized mean differences (SMDs) when measurement units differ across studies, or as mean differences (MDs) when units are consistent, each accompanied by corresponding 95% confidence intervals (CIs). Categorical outcomes will be summarized using risk ratios (RRs) with 95% CIs. When only the standard error (SE) is available, it will be converted to SD using formula (1):


$$\begin{array}{c} SD=SE\times \sqrt{n} \end{array}$$ (1)

When n is a random sample within a certain range of values, the median is taken as the basis for calculating the SD.

Data transformation for change-from-baseline outcomes is needed when only baseline and final values were reported, the mean change (mean(C)) will be calculated as formula (2):


$$\begin{array}{c} mean(C)=mean(F)-mean(B) \end{array}$$ (2)

Where mean(B) and mean(F) represent the baseline and final means, respectively.

The corresponding SD of change (SD(C)) will be estimated using formula (3):


$$\begin{array}{c} SD(C)=\sqrt{\left[SD(B{)}^{\text{2}}+SD(F{)}^{\text{2}}-\text{2}\times r\times SD(B)\times SD(F)\right]} \end{array}$$ (3)

Where SD(B) and SD(F) denote the baseline and final standard deviations, and r represents the correlation coefficient between the baseline and final measurements. If the correlation coefficient is not available, an imputed value of r = 0.5 will be assumed, in line with the Cochrane Handbook for Systematic Reviews of Interventions [40].

These transformations allow all trials to contribute comparable estimates of mean change and corresponding variability to the network meta-analysis.

If multiple doses of the same drug are reported as separate intervention arms, they will be combined. For binary variables, the sample size of each subgroup and the number of cases with the target event will be added together.

For continuous variables, formula (4) will be used for calculation:


$$\begin{array}{c} Mean=\frac{\left(N_{\text{1}}M_{\text{1}}+N_{\text{2}}M_{\text{2}}\right)}{\left(N_{\text{1}}+N_{\text{2}}\right)} \end{array}$$ (4)

The pooled standard deviation will be calculated as follows:


$$\begin{array}{c} SD=\sqrt{\frac{\left(N_{\text{1}}-\text{1}\right){SD}_{\text{1}}^{\text{2}}+\left(N_{\text{2}}-\text{1}\right){SD}_{\text{2}}^{\text{2}}+\frac{N_{\text{1}}N_{\text{2}}}{N_{\text{1}}+N_{\text{2}}}(M_{\text{1}}^{\text{2}}+M_{\text{2}}^{\text{2}}-\text{2}M_{\text{1}}M_{\text{2}})}{N_{\text{1}}+N_{\text{2}}-\text{1}}} \end{array}$$ (5)

For multiple subgroups of data that need to be combined, according to the above formula, the data of two of the subgroups will be combined first, followed by the third subgroup, and so on.

### 2.9. Methods for NMA

Network meta-analysis will be performed using Stata MP (version 18.0; StataCorp LLC, College Station, TX, USA), with additional analyses conducted in R (version 4.5.2; R Foundation for Statistical Computing, Vienna, Austria). All statistical procedures will adhere to the Cochrane Handbook, and the study will be reported in accordance with the PRISMA guidelines.

#### 2.9.1. Heterogeneity and inconsistency assessment.

Heterogeneity will be evaluated using the Cochrane Q test and I^2^ statistic. An I^2^ value < 25% will be defined as negligible heterogeneity, 25% to 49% as low heterogeneity, 50% to 74% as substantial heterogeneity, and ≥ 75% as considerable heterogeneity. A fixed-effect model will be applied when heterogeneity is negligible (I^2^ < 25%); otherwise, a random-effect model will be applied.

Global inconsistency of the entire evidence network will be assessed using the design-by-treatment interaction model, with the chi-squared (χ^2^) statistic used to test for significant discrepancy between direct and indirect comparisons [41]. A *P*-value (Prob>χ^2^) will be considered indicative of statistically significant global inconsistency. For closed loops in the evidence network, node-splitting analyses will be conducted to evaluate local inconsistency [42].

#### 2.9.2. Transitivity assessment.

The transitivity assumption will be systematically verified by comparing the distribution of pre-specified clinical and methodological effect modifiers across all treatment comparisons in the network. These modifiers include: baseline age, sex, body mass index, baseline LDL-cholesterol level, presence of established cardiovascular disease, background statin therapy, type of diabetes, and duration of follow-up. We will first perform a qualitative comparison of the distribution of these modifiers across intervention groups, followed by quantitative statistical tests (one-way ANOVA for continuous variables, chi-square test for categorical variables) to assess the balance of baseline characteristics. This assessment will identify potential imbalances in patient populations, especially between high- and low-dose trials, to verify the similarity of populations across different comparisons. If significant imbalances are detected, pre-specified subgroup analyses and meta-regression will be conducted to explore the influence of these variables on treatment effects, with corresponding sensitivity analyses performed to verify the robustness of the primary results. The potential impact of residual unadjustable imbalance on study findings will be explicitly discussed in the limitation section of the final manuscript.

#### 2.9.3. Multivariate network meta-analysis.

Network meta-analyses of RCTs will be conducted using the mvmeta command to perform multivariate network meta-analysis [43]. The surface under the cumulative ranking curve (SUCRA) will be computed to estimate the probability ranking of each intervention across outcomes [44]. A SUCRA value of 100% indicates the most effective intervention, whereas a value of 0 denotes the least effective. League tables will be generated to present pairwise comparisons between all treatments, and forest plots will be created using the interval plot command to display pooled estimates with 95% CIs. To explore the potential impact of drug dose on treatment efficacy, a dose-response analysis will be performed using the netdose package in R [45]. Dose-response curves will be generated for each lipid-modifying agent to visualize the change in treatment efficacy across the full range of observed doses.

A series of sensitivity analyses will be performed to assess the robustness of the findings, including: (1) leave-one-out sensitivity analyses; (2) re-analyses excluding studies with small sample sizes or high risk of bias; and (3) a hypertriglyceridemia-specific sensitivity analysis. Given that the accuracy of the Friedewald equation may be reduced in the presence of elevated triglyceride concentrations, we will conduct a sensitivity analysis excluding studies using Friedewald-derived LDL-C values in populations with markedly elevated triglyceride levels (e.g., a mean triglyceride level >200 mg/dL).

Potential small-study effects will be visually assessed using comparison-adjusted funnel plots when at least ten RCTs are available for a given outcome. Egger’s linear regression test will be further performed to quantitatively evaluate the asymmetry of funnel plots. All statistical tests will be two-tailed, with *P* ≤ 0.05 considered statistically significant.

#### 2.9.4. Subgroup analysis and meta-regression.

For continuous variables including baseline LDL-cholesterol level, duration of follow-up, baseline age, and body mass index, random-effects network meta-regression will be performed. For categorical variables including presence of established cardiovascular disease, background statin therapy, type of diabetes, and sex, subgroup analyses will be conducted to compare the consistency of pooled effect estimates across subgroups.

### 2.10. Strength of evidence

The CINeMA (Confidence in Network Meta-Analysis) web application will be applied to assess the certainty of direct, indirect, and network estimates for all outcomes across six domains: within-study bias, reporting bias, indirectness, imprecision, heterogeneity, and inconsistency [46,47]. Moreover, two reviewers (XZ and CT) will independently evaluate the strength of evidence for each outcome. Any disagreement at this stage will be discussed and resolved by a third reviewer (SW). The summary table of the quality of all included RCTs will be presented following the PRISMA principle.

### 2.11. Ethics and patient involvement

Patient and public involvement is not applicable to this study because it is a systematic review and network meta-analysis based exclusively on published data. Ethical approval and informed consent are not required.

### 2.12. Study status and timeline

At the time of submission, no data extraction, quantitative synthesis, or analysis has yet been performed, and no results have been generated. The planned timeline of the study is as follows:

A Participant recruitment: Not applicable, as this study is based exclusively on previously published and publicly available data.
B Data collection: The systematic literature search is expected to be completed by early January 2026.
C Record screening: Expected to be completed by late January 2026.
D Data extraction and risk-of-bias assessment: Expected to be completed by February 2026.
E Results analysis: Data synthesis, network meta-analysis, and certainty-of-evidence assessment are expected to be completed by early March 2026, with submission of the final results manuscript anticipated shortly thereafter.

Any deviations from this planned timeline or amendments to the protocol will be documented and reported in the final publication.

## 3. Discussion

Patients with diabetes experience a higher incidence of cardiovascular events compared with the general population, making the reduction of such events a primary objective in diabetes management. Given that diabetic individuals are frequent users of lipid-modifying therapies, a NMA is warranted to comprehensively evaluate the efficacy and safety of these agents across the diabetic population. In addition, subgroup analyses based on age, sex, BMI, and diabetes duration will be conducted, comparing direct and indirect evidence to further inform personalized treatment strategies. The findings of this study are intended to guide healthcare providers, policymakers, and patients and their families regarding the relative effectiveness of both traditional and novel lipid-modifying therapies.

This NMA offers several strengths. To our knowledge, it represents the most comprehensive and up-to-date systematic review and network meta-analysis assessing nearly all available lipid-modifying agents in patients with diabetes. Given the growing global prevalence of diabetes and the substantial body of evidence from prior trials, the results are expected to provide detailed, nuanced insights to inform clinical decision-making and supplement current guideline recommendations. Furthermore, the use of the CINeMA framework will enhance the reliability of the conclusions by systematically evaluating the certainty of evidence across key domains, thereby strengthening the overall robustness and credibility of the findings.

Several limitations should be acknowledged. First, variations in the definitions of cardiovascular outcomes across trials may introduce heterogeneity, and this will be mitigated by carefully reviewing outcome definitions to exclude outcomes with limited clinical relevance in the statistics. Second, not all interventions are expected to report all anticipated outcomes, which may constrain the ability to comprehensively evaluate cardiovascular effects across all lipid-modifying agents. Third, a limited number of eligible studies may restrict the feasibility of subgroup analyses, potentially limiting insights into population-specific treatment effects. A similar constraint may apply to meta-regression analyses, thereby affecting the identification of the potential sources of heterogeneity.

## Supporting information

S1 Text Search strategy. (DOCX)

S2 Text PRISMA-P-checklist. (DOCX)

## Abbreviations

ACLi: Adenosine Triphosphate-Citrate Lyase Inhibitors
ADA: American Diabetes Association
ACEIs: Angiotensin-Converting Enzyme Inhibitors
ApoB: Apolipoprotein B
ARBs: Angiotensin II Receptor Blockers
ASCVD: Atherosclerotic Cardiovascular Disease
BETi: Bromodomain and Extra-Terminal Motif Inhibitors
BMI: Body Mass Index
BNP: B-type Natriuretic Peptide
CCBs: Calcium Channel Blockers
CENTRAL: Cochrane Central Register of Controlled Trials
CINeMA: Confidence in Network Meta-Analysis
DM: Diabetes Mellitus
DPP-4i: Dipeptidyl Peptidase-4 Inhibitors
EASD: European Association for the Study of Diabetes
EMA: European Medicines Agency
FDA: U.S. Food and Drug Administration
GLP-1 RAs: Glucagon-Like Peptide-1 Receptor Agonists
HDL-C: High-Density Lipoprotein Cholesterol
hs-cTnI: high-sensitivity cardiac troponin I
hs-cTnT: high-sensitivity cardiac troponin T
IQR: Interquartile Range
LDL-C: Low-Density Lipoprotein Cholesterol
MACE: Major Adverse Cardiovascular Events
MeSH: Medical Subject Headings
NMA: Network Meta-Analysis
NT-proBNP: N-terminal pro–B-type natriuretic peptide
PCSK9i: Proprotein Convertase Subtilisin/Kexin Type 9 Inhibitors
PRISMA: Preferred Reporting Items for Systematic Reviews and Meta-Analyses
PRISMA-NMA: PRISMA extension statement for Network Meta-Analyses
PRISMA-P: Preferred Reporting Items for Systematic Review and Meta-Analysis Protocols
PROSPERO: International Prospective Register of Systematic Reviews
RCT: Randomized Controlled Trial
RoB 2.0: Cochrane Risk of Bias 2.0
RR: Risk Ratio
SGLT2i: Sodium-Glucose Cotransporter-2 Inhibitors
SD: Standard Deviation
SE: Standard Error
SMD: Standardized Mean Difference
SUCRA: Surface Under the Cumulative Ranking Curve
TC: Total Cholesterol
TG: Triglycerides.
