# Supplementary material for: Lipid-modifying pharmacotherapy in diabetes mellitus: A protocol for a systematic review and network meta-analysis of randomised trials
Source: PLoS One. 2026 Jul 23;21(7):e0354259. doi: 10.1371/journal.pone.0354259 (PMC13395341; doi:10.1371/journal.pone.0354259)
Supplement: S1 Text — (DOCX) [file pone.0354259.s001.docx]

**Supplementary materials**

**Lipid-modifying pharmacotherapy in diabetes mellitus: A protocol for a systematic review and network meta-analysis of randomised trials**

Lipid-modifying therapy in diabetes

Xudong Zhao^1,¶^, Cheng Tang^1,¶^, Tianqi Yuan^2^, Jiafan Chen^3^, Cuijuan Shi^4,5^, Xiaoyu Liu^1^, Ruigeng Yang^1^, Yushang Zhi^6^, Wenrui Huang^7^, Chunyan Zhu^8^, Yu Chen^1,*^, Shidong Wang^1,*^

Table of Contents

[Appendix 1: Search strategy of randomized controlled trial 2](#_Toc213083334)

[Appendix 2: Search strategy of meta-analysis 9](#_Toc213083335)

Appendix 1: Search strategy of randomized controlled trial

Table S1 Search strategy of PubMed

| # | searches |
| --- | --- |
| 1 | ((((((((Diabetes Mellitus[MeSH Terms]) OR (Diabetes[Title/Abstract])) OR (DM[Title/Abstract])) OR (T2DM[Title/Abstract])) OR (NIDDM[Title/Abstract])) OR (Adult-Onset Diabetes Mellitus[Title/Abstract])) OR (Ketosis-Resistant Diabetes Mellitus[Title/Abstract])) OR (Slow-Onset Diabetes Mellitus[Title/Abstract])) OR (Noninsulin Dependent Diabetes Mellitus[Title/Abstract]) |
| 2 | ((((((((((((Prediabetic State[MeSH Terms]) OR (Prediabetic State*[Title/Abstract])) OR (State*, Prediabetic[Title/Abstract])) OR (Prediabet*[Title/Abstract])) OR (IFG[Title/Abstract])) OR (impaired FPG[Title/Abstract])) OR (glucose intolerance[Title/Abstract])) OR (IGT[Title/Abstract])) OR (impaired glucose[Title/Abstract])) OR (Hyperglycemia[MeSH Terms])) OR (Hyperglycemia*[Title/Abstract])) OR (Hyperglycemia*, Postprandial[Title/Abstract])) OR (Postprandial Hyperglycemia*[Title/Abstract]) |
| 3 | #1 OR #2 |
| 4 | (((((((((((((Randomized Controlled Trial [Publication Type]) OR (Randomized Controlled Trials as Topic[MeSH Terms])) OR (Clinical Trial*, Randomized[Title/Abstract])) OR (Trial*, Randomized Clinical[Title/Abstract])) OR (Controlled Clinical Trial*, Randomized[Title/Abstract])) OR (Clinical Trial*[Title/Abstract])) OR (Controlled Clinical Trial [Publication Type])) OR (Random Allocation[MeSH Terms])) OR (Allocation, Random[Title/Abstract])) OR (Randomization[Title/Abstract])) OR (Clinical Trials as Topic[MeSH Terms])) OR (clinical trial[Publication Type])) OR (Intervention Study[Title/Abstract])) OR ((((singl*[Title/Abstract]) OR (doubl*[Title/Abstract])) OR (trebl*[Title/Abstract])) OR (tripl*[Title/Abstract])) |
| 5 | (((((((((((Review Literature as Topic[MeSH Terms]) OR (State-of-the-Art Review[Title/Abstract])) OR (State of the Art Review[Title/Abstract])) OR (State-of-the-Art Review*[Title/Abstract])) OR (Review*, State-of-the-Art[Title/Abstract])) OR (State of the Art Review*[Title/Abstract])) OR (Review [Publication Type])) OR (Review, Academic[Title/Abstract])) OR (Review, Multicase[Title/Abstract])) OR (Review of Reported Cases[Title/Abstract])) OR (Review Literature[Title/Abstract])) OR (review[Title/Abstract]) |
| 6 | (((((((Meta-Analysis[Publication Type]) OR (Meta-Analysis as Topic[MeSH Terms])) OR (Meta Analysis [Title/Abstract])) OR (Clinical Trial Overview*[Title/Abstract])) OR (Overview*, Clinical Trial[Title/Abstract])) OR ((meta analysis[MeSH Terms]) OR (metaanalysis[Title/Abstract]))) OR (((((Network Meta-Analysis[Publication Type]) OR (Network Meta-Analysis as Topic[MeSH Terms])) OR (Mixed Treatment Meta-Analysis[Title/Abstract])) OR (Multiple Treatment Comparison Meta-Analysis[Title/Abstract])) OR (Network Meta-Analysis[Title/Abstract]))) OR (((((((Systematic Review [Publication Type]) OR (Review, Systematic[Title/Abstract])) OR (Umbrella Review[Title/Abstract])) OR (Systematic Reviews as Topic[MeSH Terms])) OR (Reviews Systematic as Topic[Title/Abstract])) OR (Systematic Review as Topic[Title/Abstract])) OR (Umbrella Reviews as Topic[Title/Abstract])) |
| 7 | #5 OR #6 |
| 8 | ((((((((((((((((((Hypolipidemic Agents[MeSH Terms]) OR (Agent*, Hypolipidemic[Title/Abstract])) OR (Antihyperlipidemic*[Title/Abstract])) OR (Antilipemic Drug[Title/Abstract])) OR (Drug, Antilipemic[Title/Abstract])) OR (Antihyperlipemic*[Title/Abstract])) OR (Antilipemic Agent*[Title/Abstract])) OR (Agent*, Antilipemic[Title/Abstract])) OR (Antilipemic Drug*[Title/Abstract])) OR (Drug*, Antilipemic[Title/Abstract])) OR (Antilipemic*[Title/Abstract])) OR (Hypolipidemic Drug*[Title/Abstract])) OR (Drug*, Hypolipidemic[Title/Abstract])) OR (Hypolipidemic Agent[Title/Abstract])) OR (Agent, Hypolipidemic[Title/Abstract])) OR (Hypolipidemic Drug[Title/Abstract])) OR (Drug, Hypolipidemic[Title/Abstract])) OR (lipid-lowering drug[Title/Abstract]) |
| 9 | (((((((((((((((((((Hydroxymethylglutaryl-CoA Reductase Inhibitors[MeSH Terms]) OR (Hydroxymethylglutaryl CoA Reductase Inhibitor*[Title/Abstract])) OR (Inhibitor*, Hydroxymethylglutaryl-CoA Reductase[Title/Abstract])) OR (Reductase Inhibitor*, Hydroxymethylglutaryl-CoA[Title/Abstract])) OR (HMG-CoA Reductase Inhibitor*[Title/Abstract])) OR (HMG CoA Reductase Inhibitor*[Title/Abstract])) OR (Statin*[Title/Abstract])) OR (Inhibitor*, HMG-CoA Reductase[Title/Abstract])) OR (Inhibitor*, HMG CoA Reductase[Title/Abstract])) OR (Reductase Inhibitor*, HMG-CoA[Title/Abstract])) OR (Inhibitor*, Hydroxymethylglutaryl-Coenzyme A[Title/Abstract])) OR (Hydroxymethylglutaryl-Coenzyme A Inhibitor*[Title/Abstract])) OR (Inhibitor*, Hydroxymethylglutaryl Coenzyme A[Title/Abstract])) OR (Inhibitor*, Hydroxymethylglutaryl-CoA[Title/Abstract])) OR (Hydroxymethylglutaryl-CoA Inhibitor*[Title/Abstract])) OR (Inhibitor*, Hydroxymethylglutaryl CoA[Title/Abstract])) OR (Hydroxymethylglutaryl-CoA Reductase Inhibitor*[Title/Abstract])) OR (Statin*, HMG-CoA[Title/Abstract])) OR (HMG-CoA Statin*[Title/Abstract])) OR (Statin*, HMG CoA[Title/Abstract]) |
| 10 | (((((((((((((Atorvastatin[MeSH Terms]) OR ((3R,5R)-7-(2-(4-Fluorophenyl)-5-isopropyl-3-phenyl-4-(phenylcarbamoyl)-1H-pyrrol-1-yl)-3,5-dihydroxyheptanoic acid[Title/Abstract])) OR (Lipitor[Title/Abstract])) OR (Atorvastatin Calcium[Title/Abstract])) OR (Atorvastatin, Calcium Salt[Title/Abstract])) OR (Atorvastatin Calcium Anhydrous[Title/Abstract])) OR (Liptonorm[Title/Abstract])) OR (Atorvastatin Calcium Hydrate[Title/Abstract])) OR ("CI 981"[Title/Abstract])) OR (CI-981[Title/Abstract])) OR (CI981[Title/Abstract])) OR (Atorvastatin Calcium Trihydrate[Title/Abstract])) ) OR (Atorvastatin[Title/Abstract]) |
| 11 | ((((Rosuvastatin Calcium[MeSH Terms]) OR (Calcium, Rosuvastatin[Title/Abstract])) OR (Rosuvastatin[Title/Abstract])) OR (ZD4522[Title/Abstract])) OR (Crestor[Title/Abstract]) |
| 12 | ((((((Simvastatin[MeSH Terms]) OR (Zocor[Title/Abstract])) OR (MK-733[Title/Abstract])) OR (MK733[Title/Abstract])) OR ("MK 733"[Title/Abstract])) OR (Synvinolin[Title/Abstract])) OR (Simvastatin[Title/Abstract]) |
| 13 | ((((((((((((((((((((((((((((Pravastatin[MeSH Terms]) OR (Eptastatin[Title/Abstract])) OR (SQ-31000[Title/Abstract])) OR ("SQ 31000"[Title/Abstract])) OR (SQ-31,000[Title/Abstract])) OR ("SQ 31,000"[Title/Abstract])) OR (SQ31,000[Title/Abstract])) OR (Pravastatin tert-Octylamine Salt[Title/Abstract])) OR (Pravastatin tert Octylamine Salt[Title/Abstract])) OR (Pravastatin Sodium[Title/Abstract])) OR (Sodium Salt, Pravastatin[Title/Abstract])) OR (Pravastatin, (6 beta)-Isomer[Title/Abstract])) OR (Pravastatin Monosodium Salt, (6 beta)-Isomer[Title/Abstract])) OR (CS-514[Title/Abstract])) OR (CS514[Title/Abstract])) OR ("CS 514"[Title/Abstract])) OR (Pravachol[Title/Abstract])) OR (Pravacol[Title/Abstract])) OR (Lipostat[Title/Abstract])) OR (Elisor[Title/Abstract])) OR (RMS-431[Title/Abstract])) OR (RMS431[Title/Abstract])) OR ("RMS 431"[Title/Abstract])) OR (Apo Pravastatin[Title/Abstract])) OR (Mevalotin[Title/Abstract])) OR (Lin Pravastatin[Title/Abstract])) OR (Vasten[Title/Abstract])) OR (Nu Pravastatin[Title/Abstract])) OR (Pravastatin[Title/Abstract]) |
| 14 | ((((((((((((Lovastatin[MeSH Terms]) OR (6 Methylcompactin[Title/Abstract])) OR (Mevinolin[Title/Abstract])) OR (Monacolin K[Title/Abstract])) OR (Lovastatin, 1 alpha-Isomer[Title/Abstract])) OR (1 alpha-Isomer Lovastatin[Title/Abstract])) OR (alpha-Isomer Lovastatin, 1[Title/Abstract])) OR (Lovastatin, 1 alpha Isomer[Title/Abstract])) OR (MK-803[Title/Abstract])) OR (MK803[Title/Abstract])) OR ("MK 803"[Title/Abstract])) OR (Mevacor[Title/Abstract])) OR (Lovastatin[Title/Abstract]) |
| 15 | (((((((((((Fluvastatin[MeSH Terms]) OR (Lescol[Title/Abstract])) OR ("XU 62-320"[Title/Abstract])) OR ("XU 62 320"[Title/Abstract])) OR ("XU 62320"[Title/Abstract])) OR (XU-62320[Title/Abstract])) OR (XU62320[Title/Abstract])) OR (Fluvastatin Sodium[Title/Abstract])) OR (Fluindostatin[Title/Abstract])) OR (Fluvastatin Sodium Salt[Title/Abstract])) OR (7-(3-(4-Fluorophenyl)-1-(1-methylethyl)-1H-indol-2-yl)-3,5-dihydroxy-6-heptenoate[Title/Abstract])) OR (Fluvastatin[Title/Abstract]) |
| 16 | (((((((((((pitavastatin[Supplementary Concept]) OR ((E,3R,5S)-7-(2-cyclopropyl-4-(4-fluorophenyl)quinolin-3-yl)-3,5-dihydroxyhept-6-enoic acid[Title/Abstract])) OR (itavastatin[Title/Abstract])) OR ("NK 104"[Title/Abstract])) OR (NK-104[Title/Abstract])) OR ("P 872441"[Title/Abstract])) OR (P-872441[Title/Abstract])) OR (pitavastatin lactone[Title/Abstract])) OR (nisvastatin[Title/Abstract])) OR (pitavastatin calcium[Title/Abstract])) OR (itavastatin calcium[Title/Abstract])) OR (pitavastatin[Title/Abstract]) |
| 17 | #10 OR #11 OR #12 OR #13 OR #14 OR #15 OR #16 |
| 18 | (((((((((Ezetimibe[MeSH Terms]) OR ((1-(4-fluorophenyl[Title/Abstract])-(3R)-(3-(4-fluorophenyl)-(3S)-hydroxypropyl)-(4S)-(4-hydroxyphenyl)-2-azetidinone))) OR (Ezetimib*[Title/Abstract])) OR ("SCH 58235"[Title/Abstract])) OR (58235, SCH[Title/Abstract])) OR (SCH-58235[Title/Abstract])) OR (SCH58235[Title/Abstract])) OR (Zetia[Title/Abstract])) OR (Ezetrol[Title/Abstract])) OR (cholesterol absorption inhibitor[Title/Abstract]) |
| 19 | (((((((PCSK9 Inhibitors[MeSH Terms]) OR (PCSK9 Inhibitor*, Cardiovascular[Title/Abstract])) OR (Cardiovascular PCSK9 Inhibitor*[Title/Abstract])) OR (Inhibitor*, Cardiovascular PCSK9[Title/Abstract])) OR (PCSK9 Inhibitor* Cardiovascular[Title/Abstract])) OR (Cardiovascular, PCSK9 Inhibitor*[Title/Abstract])) OR (PCSK9 inhibitor*[Title/Abstract])) OR (Proprotein Convertase Subtilisin/Kexin Type 9[Title/Abstract]) |
| 20 | ((((((evolocumab [Supplementary Concept]) OR (repatha[Title/Abstract])) OR (AMG-145[Title/Abstract])) OR ("AMG 145"[Title/Abstract])) OR (AMG145[Title/Abstract])) OR (evolocumab[Title/Abstract])) OR ((((((((((alirocumab [Supplementary Concept]) OR (SAR236553[Title/Abstract])) OR (SAR-236553[Title/Abstract])) OR ("SAR 236553"[Title/Abstract])) OR (praluent[Title/Abstract])) OR (REGN-727[Title/Abstract])) OR (REGN727[Title/Abstract])) OR ("REGN 727"[Title/Abstract])) OR (monoclonal antibody REGN727[Title/Abstract])) OR (alirocumab[Title/Abstract])) |
| 21 | ((((((((((Fibric Acids[MeSH Terms]) OR (2-Phenoxy Isobutyric Acid*[Title/Abstract])) OR (2 Phenoxy Isobutyric Acid*[Title/Abstract])) OR (Isobutyric Acid*, 2-Phenoxy[Title/Abstract])) OR (2-Phenoxy-2-Methylpropionic Acid Derivative*[Title/Abstract])) OR (2 Phenoxy 2 Methylpropionic Acid Derivative*[Title/Abstract])) OR (Fibric Acid*[Title/Abstract])) OR (Acid Derivative*, Fibric[Title/Abstract])) OR (Methyl-2-Phenoxypropanoic Acid Derivative*[Title/Abstract])) OR (Methyl 2 Phenoxypropanoic Acid Derivative*[Title/Abstract])) OR (Fibrate*[Title/Abstract]) |
| 22 | ((((((((((((Bezafibrate[MeSH Terms]) OR (Bezafibrate[Title/Abstract])) OR (Bezalip[Title/Abstract])) OR (Cedur[Title/Abstract])) OR (BM-15.075[Title/Abstract])) OR ("BM 15.075"[Title/Abstract])) OR (Lipox[Title/Abstract])) OR (Beza Lande[Title/Abstract])) OR (Beza Puren[Title/Abstract])) OR (Bezafibrat PB[Title/Abstract])) OR (Regadrin B[Title/Abstract])) OR (Béfizal[Title/Abstract])) OR (Eulitop[Title/Abstract]) |
| 23 | ((((Clofenapate[MeSH Terms]) OR (Clofenapate[Title/Abstract])) OR (Methyl Clofenapate[Title/Abstract])) OR (Clofenapate, Methyl[Title/Abstract])) OR (Methylclofenapate[Title/Abstract]) |
| 24 | ((((((((((((((((((((((((((((((((((((((((((((((((((((((((((Fenofibrate[MeSH Terms]) OR (Fenofibrate[Title/Abstract])) OR (Procetofen[Title/Abstract])) OR (Phenofibrate[Title/Abstract])) OR (Procetofene[Title/Abstract])) OR (Lipanthyl[Title/Abstract])) OR (Fénofibrate Debat[Title/Abstract])) OR (Debat, Fénofibrate[Title/Abstract])) OR (Lipantil[Title/Abstract])) OR (Lipidil-Ter[Title/Abstract])) OR (Lipidil Ter[Title/Abstract])) OR (Lipidil[Title/Abstract])) OR (Secalip[Title/Abstract])) OR (Supralip[Title/Abstract])) OR (Fenofibrat FPh[Title/Abstract])) OR (LF-178[Title/Abstract])) OR (LF178[Title/Abstract])) OR ("LF 178"[Title/Abstract])) OR (Apo-Feno-Micro[Title/Abstract])) OR (Apo Feno Micro[Title/Abstract])) OR (Apo-Fenofibrate[Title/Abstract])) OR (Apo Fenofibrate[Title/Abstract])) OR (CiL[Title/Abstract])) OR (Controlip[Title/Abstract])) OR (durafenat[Title/Abstract])) OR (Lofibra[Title/Abstract])) OR (Fénofibrate MSD[Title/Abstract])) OR (Gen-Fenofibrate[Title/Abstract])) OR (Gen Fenofibrate[Title/Abstract])) OR (Liparison[Title/Abstract])) OR (Livesan[Title/Abstract])) OR (MTW-Fenofibrat[Title/Abstract])) OR (MTW Fenofibrat[Title/Abstract])) OR (Tricor[Title/Abstract])) OR (Normalip[Title/Abstract])) OR (Novo-Fenofibrate[Title/Abstract])) OR (Novo Fenofibrate[Title/Abstract])) OR (Nu-Fenofibrate[Title/Abstract])) OR (Nu Fenofibrate[Title/Abstract])) OR (PMS-Fenofibrate Micro[Title/Abstract])) OR (PMS Fenofibrate Micro[Title/Abstract])) OR (Fenobeta[Title/Abstract])) OR (Fenofanton[Title/Abstract])) OR (Fenofibrat AbZ[Title/Abstract])) OR (Fenofibrat AL[Title/Abstract])) OR (Fenofibrat AZU[Title/Abstract])) OR (AZU, Fenofibrat[Title/Abstract])) OR (Fenofibrat Heumann[Title/Abstract])) OR (Heumann, Fenofibrat[Title/Abstract])) OR (Fenofibrat Hexal[Title/Abstract])) OR (Hexal, Fenofibrat[Title/Abstract])) OR (Fenofibrat Stada[Title/Abstract])) OR (Stada, Fenofibrat[Title/Abstract])) OR (fenofibrat von ct[Title/Abstract])) OR (Fenofibrat-ratiopharm[Title/Abstract])) OR (Fenofibrat ratiopharm[Title/Abstract])) OR (Antara Micronized Procetofen[Title/Abstract])) OR (Micronized Procetofen, Antara[Title/Abstract])) OR (Procetofen, Antara Micronized[Title/Abstract]) |
| 25 | ((((((((((((((((((((((((((((((((((Gemfibrozil[MeSH Terms]) OR (Gemfibrozil[Title/Abstract])) OR (Gemfibrosil[Title/Abstract])) OR (Lopid[Title/Abstract])) OR (Lopid R[Title/Abstract])) OR (Lipur[Title/Abstract])) OR (CI-719[Title/Abstract])) OR (CI719[Title/Abstract])) OR ("CI 719"[Title/Abstract])) OR (Apo-Gemfibrozil[Title/Abstract])) OR (Apo Gemfibrozil[Title/Abstract])) OR (Ausgem[Title/Abstract])) OR (Bolutol[Title/Abstract])) OR (Chem mart Gemfibrozil[Title/Abstract])) OR (DBL Gemfibrozil[Title/Abstract])) OR (Decrelip[Title/Abstract])) OR (Gemfi 1A Pharma[Title/Abstract])) OR (Gemfibrozilo Ur[Title/Abstract])) OR (Gen Gemfibrozil[Title/Abstract])) OR (GenRX Gemfibrozil[Title/Abstract])) OR (Gemfibrozil, GenRX[Title/Abstract])) OR (Healthsense Gemfibrozil[Title/Abstract])) OR (Gemfibrozil, Healthsense[Title/Abstract])) OR (Jezil[Title/Abstract])) OR (Lipazil[Title/Abstract])) OR (Lipox Gemfi[Title/Abstract])) OR (Litarek[Title/Abstract])) OR (Novo Gemfibrozil[Title/Abstract])) OR (Nu Gemfibrozil[Title/Abstract])) OR (Pilder[Title/Abstract])) OR (PMS Gemfibrozil[Title/Abstract])) OR (SBPA Gemfibrozil[Title/Abstract])) OR (Gemfibrozil, SBPA[Title/Abstract])) OR (Terry White Chemists Gemfibrozil[Title/Abstract])) OR (Trialmin[Title/Abstract]) |
| 26 | #22 OR #23 OR #24 OR #25 |
| 27 | (((((((((((((((((((((((((((((((((((((Niacin[MeSH Terms]) OR (3-Pyridinecarboxylic Acid[Title/Abstract])) OR (3 Pyridinecarboxylic Acid[Title/Abstract])) OR (Nicotinic Acid[Title/Abstract])) OR (Nicotinate[Title/Abstract])) OR (Enduracin[Title/Abstract])) OR (Induracin[Title/Abstract])) OR (Nicobid[Title/Abstract])) OR (Nicamin[Title/Abstract])) OR (Nico-400[Title/Abstract])) OR (Nico400[Title/Abstract])) OR ("Nico 400"[Title/Abstract])) OR (Nicolar[Title/Abstract])) OR (Niacin Iron (2+) Salt[Title/Abstract])) OR (Niacin Lithium Salt, Hemihydrate[Title/Abstract])) OR (Niacin Potassium Salt[Title/Abstract])) OR (Potassium Salt, Niacin[Title/Abstract])) OR (Niacin Aluminum Salt[Title/Abstract])) OR (Aluminum Salt, Niacin[Title/Abstract])) OR (Niacin Cobalt (2+) Salt[Title/Abstract])) OR (Niacin Manganese (2+) Salt[Title/Abstract])) OR (Niacin Copper (2+) Salt[Title/Abstract])) OR (Niacin Ammonium Salt[Title/Abstract])) OR (Niacin Tartrate[Title/Abstract])) OR (Tartrate, Niacin[Title/Abstract])) OR (Niacin Zinc Salt[Title/Abstract])) OR (Niacin Lithium Salt[Title/Abstract])) OR (Niacin Sodium Salt[Title/Abstract])) OR (Sodium Salt, Niacin[Title/Abstract])) OR (Niacin Hydrochloride[Title/Abstract])) OR (Hydrochloride, Niacin[Title/Abstract])) OR (Niacin Magnesium Salt[Title/Abstract])) OR (Niacin Calcium Salt[Title/Abstract])) OR (Lithium Nicotinate[Title/Abstract])) OR (Nicotinate, Lithium[Title/Abstract])) OR (Niacin Tosylate[Title/Abstract])) OR (Tosylate, Niacin[Title/Abstract])) OR (Niacin[Title/Abstract]) |
| 28 | (((((((((((((((((((((((Fatty Acids, Omega-3[MeSH Terms]) OR (N-3 Fatty Acid*[Title/Abstract])) OR (Acid, N-3 Fatty[Title/Abstract])) OR (Fatty Acid, N-3[Title/Abstract])) OR (N 3 Fatty Acid[Title/Abstract])) OR (Omega-3 Fatty Acid*[Title/Abstract])) OR (Acid, Omega-3 Fatty[Title/Abstract])) OR (Fatty Acid, Omega-3[Title/Abstract])) OR (Omega 3 Fatty Acid[Title/Abstract])) OR ("n-3 Oil*"[Title/Abstract])) OR ("n 3 Oil*"[Title/Abstract])) OR (Oil, n-3[Title/Abstract])) OR (Oil, n3[Title/Abstract])) OR (n-3 Polyunsaturated Fatty Acid[Title/Abstract])) OR (n 3 Polyunsaturated Fatty Acid[Title/Abstract])) OR ("n-3 PUFA"[Title/Abstract])) OR ("n 3 PUFA"[Title/Abstract])) OR (PUFA, n-3[Title/Abstract])) OR ("n3 Fatty Acid"[Title/Abstract])) OR (Fatty Acid, n3[Title/Abstract])) OR ("n3 PUFA"[Title/Abstract])) OR (PUFA, n3[Title/Abstract])) OR (n3 Polyunsaturated Fatty Acid[Title/Abstract])) |
| 29 | ((((((Omacor [Supplementary Concept]) OR (Lovaza[Title/Abstract])) OR (omega-3 ethyl ester 90[Title/Abstract])) OR (P-OM3 adjunct[Title/Abstract])) ) OR (Omacor[Title/Abstract])) OR (((((((((((eicosapentaenoic acid ethyl ester [Supplementary Concept]) OR (icosapent ethyl[Title/Abstract])) OR (5,8,11,14,17-eicosapentaenoic acid, ethyl ester, (5Z,8Z,11Z,14Z,17Z)-[Title/Abstract])) OR (ethyl all-cis-5,8,11,14,17-icosapentaenoate[Title/Abstract])) OR (ethyl eicosapentaenoate[Title/Abstract])) OR (ethyl icosapentaenoate[Title/Abstract])) OR (ethyl eicosapentaenoic acid[Title/Abstract])) OR (ethyl-EPA[Title/Abstract])) OR (ethyl-eicosapentaenoic acid[Title/Abstract])) OR (Epadel[Title/Abstract])) OR (vascepa[Title/Abstract])) OR (AMR101[Title/Abstract]) |
| 30 | ((bile acid sequestrant*[Title/Abstract]) OR (bile-acid sequestrant[Title/Abstract])) OR (Bile acid sequestrant antilipemic agent[Title/Abstract]) |
| 31 | (((((((((((((((((((((Colesevelam Hydrochloride[MeSH Terms]) OR (Hydrochloride, Colesevelam[Title/Abstract])) OR (Colesevelam HCl[Title/Abstract])) OR (HCl, Colesevelam[Title/Abstract])) OR (Colesevelam[Title/Abstract])) OR ("GT 31104"[Title/Abstract])) OR (31104, GT[Title/Abstract])) OR (GT31-104[Title/Abstract])) OR (GT31104[Title/Abstract])) OR (GT31 104[Title/Abstract])) OR (GT-31104[Title/Abstract])) OR (CholestaGel[Title/Abstract])) OR (Welchol[Title/Abstract])) OR (Cholestyramine Resin*[MeSH Terms])) OR (Resin*, Cholestyramine[Title/Abstract])) OR (Cholestyramine*[Title/Abstract])) OR (Colestyramin*[Title/Abstract])) OR (Cuemid*[Title/Abstract])) OR (Quantalan*[Title/Abstract])) OR (Questran*[Title/Abstract])) OR (MK-135[Title/Abstract])) OR ("MK 135"[Title/Abstract]) |
| 32 | (ATP citrate lyase inhibitor[Title/Abstract]) OR (ACL inhibitor[Title/Abstract]) |
| 33 | ((((((((8-hydroxy-2,2,14,14-tetramethylpentadecanedioic acid [Supplementary Concept]) OR (bempedoic acid[Title/Abstract])) OR (ETC-1002[Title/Abstract])) OR (ESP-55016[Title/Abstract])) OR (nexletol[Title/Abstract])) OR (nilemdo[Title/Abstract])) OR ("ESP 55016"[Title/Abstract])) OR (ETC1002[Title/Abstract])) OR ("ETC 1002"[Title/Abstract]) |
| 34 | (((ALN-PCS [Supplementary Concept]) OR (ALN-PCSsc[Title/Abstract])) OR (inclisiran[Title/Abstract])) OR (leqvio[Title/Abstract]) |
| 35 | #8 OR #9 OR #17 OR #18 OR #19 OR #20 OR #21 OR #26 OR #27 OR #28 OR #29 OR #30 OR #31 OR #32 OR #33 OR #34 |
| 36 | #3 AND #4 AND #35 |
| 37 | #36 NOT #7 |

Appendix 2: Search strategy of meta-analysis

Table S2 Search strategy of PubMed

| # | searches |
| --- | --- |
| 1 | ((((((((Diabetes Mellitus[MeSH Terms]) OR (Diabetes[Title/Abstract])) OR (DM[Title/Abstract])) OR (T2DM[Title/Abstract])) OR (NIDDM[Title/Abstract])) OR (Adult-Onset Diabetes Mellitus[Title/Abstract])) OR (Ketosis-Resistant Diabetes Mellitus[Title/Abstract])) OR (Slow-Onset Diabetes Mellitus[Title/Abstract])) OR (Noninsulin Dependent Diabetes Mellitus[Title/Abstract]) |
| 2 | ((((((((((((Prediabetic State[MeSH Terms]) OR (Prediabetic State*[Title/Abstract])) OR (State*, Prediabetic[Title/Abstract])) OR (Prediabet*[Title/Abstract])) OR (IFG[Title/Abstract])) OR (impaired FPG[Title/Abstract])) OR (glucose intolerance[Title/Abstract])) OR (IGT[Title/Abstract])) OR (impaired glucose[Title/Abstract])) OR (Hyperglycemia[MeSH Terms])) OR (Hyperglycemia*[Title/Abstract])) OR (Hyperglycemia*, Postprandial[Title/Abstract])) OR (Postprandial Hyperglycemia*[Title/Abstract]) |
| 3 | #1 OR #2 |
| 4 | (((((((Meta-Analysis[Publication Type]) OR (Meta-Analysis as Topic[MeSH Terms])) OR (Meta Analysis [Title/Abstract])) OR (Clinical Trial Overview*[Title/Abstract])) OR (Overview*, Clinical Trial[Title/Abstract])) OR ((meta analysis[MeSH Terms]) OR (metaanalysis[Title/Abstract]))) |
| 5 | (((((Network Meta-Analysis[Publication Type]) OR (Network Meta-Analysis as Topic[MeSH Terms])) OR (Mixed Treatment Meta-Analysis[Title/Abstract])) OR (Multiple Treatment Comparison Meta-Analysis[Title/Abstract])) OR (Network Meta-Analysis[Title/Abstract]))) |
| 6 | (((((((Systematic Review [Publication Type]) OR (Review, Systematic[Title/Abstract])) OR (Umbrella Review[Title/Abstract])) OR (Systematic Reviews as Topic[MeSH Terms])) OR (Reviews Systematic as Topic[Title/Abstract])) OR (Systematic Review as Topic[Title/Abstract])) OR (Umbrella Reviews as Topic[Title/Abstract])) |
| 7 | #4 OR #5 OR #6 |
| 8 | ((((((((((((((((((Hypolipidemic Agents[MeSH Terms]) OR (Agent*, Hypolipidemic[Title/Abstract])) OR (Antihyperlipidemic*[Title/Abstract])) OR (Antilipemic Drug[Title/Abstract])) OR (Drug, Antilipemic[Title/Abstract])) OR (Antihyperlipemic*[Title/Abstract])) OR (Antilipemic Agent*[Title/Abstract])) OR (Agent*, Antilipemic[Title/Abstract])) OR (Antilipemic Drug*[Title/Abstract])) OR (Drug*, Antilipemic[Title/Abstract])) OR (Antilipemic*[Title/Abstract])) OR (Hypolipidemic Drug*[Title/Abstract])) OR (Drug*, Hypolipidemic[Title/Abstract])) OR (Hypolipidemic Agent[Title/Abstract])) OR (Agent, Hypolipidemic[Title/Abstract])) OR (Hypolipidemic Drug[Title/Abstract])) OR (Drug, Hypolipidemic[Title/Abstract])) OR (lipid-lowering drug[Title/Abstract]) |
| 9 | (((((((((((((((((((Hydroxymethylglutaryl-CoA Reductase Inhibitors[MeSH Terms]) OR (Hydroxymethylglutaryl CoA Reductase Inhibitor*[Title/Abstract])) OR (Inhibitor*, Hydroxymethylglutaryl-CoA Reductase[Title/Abstract])) OR (Reductase Inhibitor*, Hydroxymethylglutaryl-CoA[Title/Abstract])) OR (HMG-CoA Reductase Inhibitor*[Title/Abstract])) OR (HMG CoA Reductase Inhibitor*[Title/Abstract])) OR (Statin*[Title/Abstract])) OR (Inhibitor*, HMG-CoA Reductase[Title/Abstract])) OR (Inhibitor*, HMG CoA Reductase[Title/Abstract])) OR (Reductase Inhibitor*, HMG-CoA[Title/Abstract])) OR (Inhibitor*, Hydroxymethylglutaryl-Coenzyme A[Title/Abstract])) OR (Hydroxymethylglutaryl-Coenzyme A Inhibitor*[Title/Abstract])) OR (Inhibitor*, Hydroxymethylglutaryl Coenzyme A[Title/Abstract])) OR (Inhibitor*, Hydroxymethylglutaryl-CoA[Title/Abstract])) OR (Hydroxymethylglutaryl-CoA Inhibitor*[Title/Abstract])) OR (Inhibitor*, Hydroxymethylglutaryl CoA[Title/Abstract])) OR (Hydroxymethylglutaryl-CoA Reductase Inhibitor*[Title/Abstract])) OR (Statin*, HMG-CoA[Title/Abstract])) OR (HMG-CoA Statin*[Title/Abstract])) OR (Statin*, HMG CoA[Title/Abstract]) |
| 10 | (((((((((((((Atorvastatin[MeSH Terms]) OR ((3R,5R)-7-(2-(4-Fluorophenyl)-5-isopropyl-3-phenyl-4-(phenylcarbamoyl)-1H-pyrrol-1-yl)-3,5-dihydroxyheptanoic acid[Title/Abstract])) OR (Lipitor[Title/Abstract])) OR (Atorvastatin Calcium[Title/Abstract])) OR (Atorvastatin, Calcium Salt[Title/Abstract])) OR (Atorvastatin Calcium Anhydrous[Title/Abstract])) OR (Liptonorm[Title/Abstract])) OR (Atorvastatin Calcium Hydrate[Title/Abstract])) OR ("CI 981"[Title/Abstract])) OR (CI-981[Title/Abstract])) OR (CI981[Title/Abstract])) OR (Atorvastatin Calcium Trihydrate[Title/Abstract])) ) OR (Atorvastatin[Title/Abstract]) |
| 11 | ((((Rosuvastatin Calcium[MeSH Terms]) OR (Calcium, Rosuvastatin[Title/Abstract])) OR (Rosuvastatin[Title/Abstract])) OR (ZD4522[Title/Abstract])) OR (Crestor[Title/Abstract]) |
| 12 | ((((((Simvastatin[MeSH Terms]) OR (Zocor[Title/Abstract])) OR (MK-733[Title/Abstract])) OR (MK733[Title/Abstract])) OR ("MK 733"[Title/Abstract])) OR (Synvinolin[Title/Abstract])) OR (Simvastatin[Title/Abstract]) |
| 13 | ((((((((((((((((((((((((((((Pravastatin[MeSH Terms]) OR (Eptastatin[Title/Abstract])) OR (SQ-31000[Title/Abstract])) OR ("SQ 31000"[Title/Abstract])) OR (SQ-31,000[Title/Abstract])) OR ("SQ 31,000"[Title/Abstract])) OR (SQ31,000[Title/Abstract])) OR (Pravastatin tert-Octylamine Salt[Title/Abstract])) OR (Pravastatin tert Octylamine Salt[Title/Abstract])) OR (Pravastatin Sodium[Title/Abstract])) OR (Sodium Salt, Pravastatin[Title/Abstract])) OR (Pravastatin, (6 beta)-Isomer[Title/Abstract])) OR (Pravastatin Monosodium Salt, (6 beta)-Isomer[Title/Abstract])) OR (CS-514[Title/Abstract])) OR (CS514[Title/Abstract])) OR ("CS 514"[Title/Abstract])) OR (Pravachol[Title/Abstract])) OR (Pravacol[Title/Abstract])) OR (Lipostat[Title/Abstract])) OR (Elisor[Title/Abstract])) OR (RMS-431[Title/Abstract])) OR (RMS431[Title/Abstract])) OR ("RMS 431"[Title/Abstract])) OR (Apo Pravastatin[Title/Abstract])) OR (Mevalotin[Title/Abstract])) OR (Lin Pravastatin[Title/Abstract])) OR (Vasten[Title/Abstract])) OR (Nu Pravastatin[Title/Abstract])) OR (Pravastatin[Title/Abstract]) |
| 14 | ((((((((((((Lovastatin[MeSH Terms]) OR (6 Methylcompactin[Title/Abstract])) OR (Mevinolin[Title/Abstract])) OR (Monacolin K[Title/Abstract])) OR (Lovastatin, 1 alpha-Isomer[Title/Abstract])) OR (1 alpha-Isomer Lovastatin[Title/Abstract])) OR (alpha-Isomer Lovastatin, 1[Title/Abstract])) OR (Lovastatin, 1 alpha Isomer[Title/Abstract])) OR (MK-803[Title/Abstract])) OR (MK803[Title/Abstract])) OR ("MK 803"[Title/Abstract])) OR (Mevacor[Title/Abstract])) OR (Lovastatin[Title/Abstract]) |
| 15 | (((((((((((Fluvastatin[MeSH Terms]) OR (Lescol[Title/Abstract])) OR ("XU 62-320"[Title/Abstract])) OR ("XU 62 320"[Title/Abstract])) OR ("XU 62320"[Title/Abstract])) OR (XU-62320[Title/Abstract])) OR (XU62320[Title/Abstract])) OR (Fluvastatin Sodium[Title/Abstract])) OR (Fluindostatin[Title/Abstract])) OR (Fluvastatin Sodium Salt[Title/Abstract])) OR (7-(3-(4-Fluorophenyl)-1-(1-methylethyl)-1H-indol-2-yl)-3,5-dihydroxy-6-heptenoate[Title/Abstract])) OR (Fluvastatin[Title/Abstract]) |
| 16 | (((((((((((pitavastatin[Supplementary Concept]) OR ((E,3R,5S)-7-(2-cyclopropyl-4-(4-fluorophenyl)quinolin-3-yl)-3,5-dihydroxyhept-6-enoic acid[Title/Abstract])) OR (itavastatin[Title/Abstract])) OR ("NK 104"[Title/Abstract])) OR (NK-104[Title/Abstract])) OR ("P 872441"[Title/Abstract])) OR (P-872441[Title/Abstract])) OR (pitavastatin lactone[Title/Abstract])) OR (nisvastatin[Title/Abstract])) OR (pitavastatin calcium[Title/Abstract])) OR (itavastatin calcium[Title/Abstract])) OR (pitavastatin[Title/Abstract]) |
| 17 | #10 OR #11 OR #12 OR #13 OR #14 OR #15 OR #16 |
| 18 | (((((((((Ezetimibe[MeSH Terms]) OR ((1-(4-fluorophenyl[Title/Abstract])-(3R)-(3-(4-fluorophenyl)-(3S)-hydroxypropyl)-(4S)-(4-hydroxyphenyl)-2-azetidinone))) OR (Ezetimib*[Title/Abstract])) OR ("SCH 58235"[Title/Abstract])) OR (58235, SCH[Title/Abstract])) OR (SCH-58235[Title/Abstract])) OR (SCH58235[Title/Abstract])) OR (Zetia[Title/Abstract])) OR (Ezetrol[Title/Abstract])) OR (cholesterol absorption inhibitor[Title/Abstract]) |
| 19 | (((((((PCSK9 Inhibitors[MeSH Terms]) OR (PCSK9 Inhibitor*, Cardiovascular[Title/Abstract])) OR (Cardiovascular PCSK9 Inhibitor*[Title/Abstract])) OR (Inhibitor*, Cardiovascular PCSK9[Title/Abstract])) OR (PCSK9 Inhibitor* Cardiovascular[Title/Abstract])) OR (Cardiovascular, PCSK9 Inhibitor*[Title/Abstract])) OR (PCSK9 inhibitor*[Title/Abstract])) OR (Proprotein Convertase Subtilisin/Kexin Type 9[Title/Abstract]) |
| 20 | ((((((evolocumab [Supplementary Concept]) OR (repatha[Title/Abstract])) OR (AMG-145[Title/Abstract])) OR ("AMG 145"[Title/Abstract])) OR (AMG145[Title/Abstract])) OR (evolocumab[Title/Abstract])) OR ((((((((((alirocumab [Supplementary Concept]) OR (SAR236553[Title/Abstract])) OR (SAR-236553[Title/Abstract])) OR ("SAR 236553"[Title/Abstract])) OR (praluent[Title/Abstract])) OR (REGN-727[Title/Abstract])) OR (REGN727[Title/Abstract])) OR ("REGN 727"[Title/Abstract])) OR (monoclonal antibody REGN727[Title/Abstract])) OR (alirocumab[Title/Abstract])) |
| 21 | ((((((((((Fibric Acids[MeSH Terms]) OR (2-Phenoxy Isobutyric Acid*[Title/Abstract])) OR (2 Phenoxy Isobutyric Acid*[Title/Abstract])) OR (Isobutyric Acid*, 2-Phenoxy[Title/Abstract])) OR (2-Phenoxy-2-Methylpropionic Acid Derivative*[Title/Abstract])) OR (2 Phenoxy 2 Methylpropionic Acid Derivative*[Title/Abstract])) OR (Fibric Acid*[Title/Abstract])) OR (Acid Derivative*, Fibric[Title/Abstract])) OR (Methyl-2-Phenoxypropanoic Acid Derivative*[Title/Abstract])) OR (Methyl 2 Phenoxypropanoic Acid Derivative*[Title/Abstract])) OR (Fibrate*[Title/Abstract]) |
| 22 | ((((((((((((Bezafibrate[MeSH Terms]) OR (Bezafibrate[Title/Abstract])) OR (Bezalip[Title/Abstract])) OR (Cedur[Title/Abstract])) OR (BM-15.075[Title/Abstract])) OR ("BM 15.075"[Title/Abstract])) OR (Lipox[Title/Abstract])) OR (Beza Lande[Title/Abstract])) OR (Beza Puren[Title/Abstract])) OR (Bezafibrat PB[Title/Abstract])) OR (Regadrin B[Title/Abstract])) OR (Béfizal[Title/Abstract])) OR (Eulitop[Title/Abstract]) |
| 23 | ((((Clofenapate[MeSH Terms]) OR (Clofenapate[Title/Abstract])) OR (Methyl Clofenapate[Title/Abstract])) OR (Clofenapate, Methyl[Title/Abstract])) OR (Methylclofenapate[Title/Abstract]) |
| 24 | ((((((((((((((((((((((((((((((((((((((((((((((((((((((((((Fenofibrate[MeSH Terms]) OR (Fenofibrate[Title/Abstract])) OR (Procetofen[Title/Abstract])) OR (Phenofibrate[Title/Abstract])) OR (Procetofene[Title/Abstract])) OR (Lipanthyl[Title/Abstract])) OR (Fénofibrate Debat[Title/Abstract])) OR (Debat, Fénofibrate[Title/Abstract])) OR (Lipantil[Title/Abstract])) OR (Lipidil-Ter[Title/Abstract])) OR (Lipidil Ter[Title/Abstract])) OR (Lipidil[Title/Abstract])) OR (Secalip[Title/Abstract])) OR (Supralip[Title/Abstract])) OR (Fenofibrat FPh[Title/Abstract])) OR (LF-178[Title/Abstract])) OR (LF178[Title/Abstract])) OR ("LF 178"[Title/Abstract])) OR (Apo-Feno-Micro[Title/Abstract])) OR (Apo Feno Micro[Title/Abstract])) OR (Apo-Fenofibrate[Title/Abstract])) OR (Apo Fenofibrate[Title/Abstract])) OR (CiL[Title/Abstract])) OR (Controlip[Title/Abstract])) OR (durafenat[Title/Abstract])) OR (Lofibra[Title/Abstract])) OR (Fénofibrate MSD[Title/Abstract])) OR (Gen-Fenofibrate[Title/Abstract])) OR (Gen Fenofibrate[Title/Abstract])) OR (Liparison[Title/Abstract])) OR (Livesan[Title/Abstract])) OR (MTW-Fenofibrat[Title/Abstract])) OR (MTW Fenofibrat[Title/Abstract])) OR (Tricor[Title/Abstract])) OR (Normalip[Title/Abstract])) OR (Novo-Fenofibrate[Title/Abstract])) OR (Novo Fenofibrate[Title/Abstract])) OR (Nu-Fenofibrate[Title/Abstract])) OR (Nu Fenofibrate[Title/Abstract])) OR (PMS-Fenofibrate Micro[Title/Abstract])) OR (PMS Fenofibrate Micro[Title/Abstract])) OR (Fenobeta[Title/Abstract])) OR (Fenofanton[Title/Abstract])) OR (Fenofibrat AbZ[Title/Abstract])) OR (Fenofibrat AL[Title/Abstract])) OR (Fenofibrat AZU[Title/Abstract])) OR (AZU, Fenofibrat[Title/Abstract])) OR (Fenofibrat Heumann[Title/Abstract])) OR (Heumann, Fenofibrat[Title/Abstract])) OR (Fenofibrat Hexal[Title/Abstract])) OR (Hexal, Fenofibrat[Title/Abstract])) OR (Fenofibrat Stada[Title/Abstract])) OR (Stada, Fenofibrat[Title/Abstract])) OR (fenofibrat von ct[Title/Abstract])) OR (Fenofibrat-ratiopharm[Title/Abstract])) OR (Fenofibrat ratiopharm[Title/Abstract])) OR (Antara Micronized Procetofen[Title/Abstract])) OR (Micronized Procetofen, Antara[Title/Abstract])) OR (Procetofen, Antara Micronized[Title/Abstract]) |
| 25 | ((((((((((((((((((((((((((((((((((Gemfibrozil[MeSH Terms]) OR (Gemfibrozil[Title/Abstract])) OR (Gemfibrosil[Title/Abstract])) OR (Lopid[Title/Abstract])) OR (Lopid R[Title/Abstract])) OR (Lipur[Title/Abstract])) OR (CI-719[Title/Abstract])) OR (CI719[Title/Abstract])) OR ("CI 719"[Title/Abstract])) OR (Apo-Gemfibrozil[Title/Abstract])) OR (Apo Gemfibrozil[Title/Abstract])) OR (Ausgem[Title/Abstract])) OR (Bolutol[Title/Abstract])) OR (Chem mart Gemfibrozil[Title/Abstract])) OR (DBL Gemfibrozil[Title/Abstract])) OR (Decrelip[Title/Abstract])) OR (Gemfi 1A Pharma[Title/Abstract])) OR (Gemfibrozilo Ur[Title/Abstract])) OR (Gen Gemfibrozil[Title/Abstract])) OR (GenRX Gemfibrozil[Title/Abstract])) OR (Gemfibrozil, GenRX[Title/Abstract])) OR (Healthsense Gemfibrozil[Title/Abstract])) OR (Gemfibrozil, Healthsense[Title/Abstract])) OR (Jezil[Title/Abstract])) OR (Lipazil[Title/Abstract])) OR (Lipox Gemfi[Title/Abstract])) OR (Litarek[Title/Abstract])) OR (Novo Gemfibrozil[Title/Abstract])) OR (Nu Gemfibrozil[Title/Abstract])) OR (Pilder[Title/Abstract])) OR (PMS Gemfibrozil[Title/Abstract])) OR (SBPA Gemfibrozil[Title/Abstract])) OR (Gemfibrozil, SBPA[Title/Abstract])) OR (Terry White Chemists Gemfibrozil[Title/Abstract])) OR (Trialmin[Title/Abstract]) |
| 26 | #22 OR #23 OR #24 OR #25 |
| 27 | (((((((((((((((((((((((((((((((((((((Niacin[MeSH Terms]) OR (3-Pyridinecarboxylic Acid[Title/Abstract])) OR (3 Pyridinecarboxylic Acid[Title/Abstract])) OR (Nicotinic Acid[Title/Abstract])) OR (Nicotinate[Title/Abstract])) OR (Enduracin[Title/Abstract])) OR (Induracin[Title/Abstract])) OR (Nicobid[Title/Abstract])) OR (Nicamin[Title/Abstract])) OR (Nico-400[Title/Abstract])) OR (Nico400[Title/Abstract])) OR ("Nico 400"[Title/Abstract])) OR (Nicolar[Title/Abstract])) OR (Niacin Iron (2+) Salt[Title/Abstract])) OR (Niacin Lithium Salt, Hemihydrate[Title/Abstract])) OR (Niacin Potassium Salt[Title/Abstract])) OR (Potassium Salt, Niacin[Title/Abstract])) OR (Niacin Aluminum Salt[Title/Abstract])) OR (Aluminum Salt, Niacin[Title/Abstract])) OR (Niacin Cobalt (2+) Salt[Title/Abstract])) OR (Niacin Manganese (2+) Salt[Title/Abstract])) OR (Niacin Copper (2+) Salt[Title/Abstract])) OR (Niacin Ammonium Salt[Title/Abstract])) OR (Niacin Tartrate[Title/Abstract])) OR (Tartrate, Niacin[Title/Abstract])) OR (Niacin Zinc Salt[Title/Abstract])) OR (Niacin Lithium Salt[Title/Abstract])) OR (Niacin Sodium Salt[Title/Abstract])) OR (Sodium Salt, Niacin[Title/Abstract])) OR (Niacin Hydrochloride[Title/Abstract])) OR (Hydrochloride, Niacin[Title/Abstract])) OR (Niacin Magnesium Salt[Title/Abstract])) OR (Niacin Calcium Salt[Title/Abstract])) OR (Lithium Nicotinate[Title/Abstract])) OR (Nicotinate, Lithium[Title/Abstract])) OR (Niacin Tosylate[Title/Abstract])) OR (Tosylate, Niacin[Title/Abstract])) OR (Niacin[Title/Abstract]) |
| 28 | (((((((((((((((((((((((Fatty Acids, Omega-3[MeSH Terms]) OR (N-3 Fatty Acid*[Title/Abstract])) OR (Acid, N-3 Fatty[Title/Abstract])) OR (Fatty Acid, N-3[Title/Abstract])) OR (N 3 Fatty Acid[Title/Abstract])) OR (Omega-3 Fatty Acid*[Title/Abstract])) OR (Acid, Omega-3 Fatty[Title/Abstract])) OR (Fatty Acid, Omega-3[Title/Abstract])) OR (Omega 3 Fatty Acid[Title/Abstract])) OR ("n-3 Oil*"[Title/Abstract])) OR ("n 3 Oil*"[Title/Abstract])) OR (Oil, n-3[Title/Abstract])) OR (Oil, n3[Title/Abstract])) OR (n-3 Polyunsaturated Fatty Acid[Title/Abstract])) OR (n 3 Polyunsaturated Fatty Acid[Title/Abstract])) OR ("n-3 PUFA"[Title/Abstract])) OR ("n 3 PUFA"[Title/Abstract])) OR (PUFA, n-3[Title/Abstract])) OR ("n3 Fatty Acid"[Title/Abstract])) OR (Fatty Acid, n3[Title/Abstract])) OR ("n3 PUFA"[Title/Abstract])) OR (PUFA, n3[Title/Abstract])) OR (n3 Polyunsaturated Fatty Acid[Title/Abstract])) |
| 29 | ((((((Omacor [Supplementary Concept]) OR (Lovaza[Title/Abstract])) OR (omega-3 ethyl ester 90[Title/Abstract])) OR (P-OM3 adjunct[Title/Abstract])) ) OR (Omacor[Title/Abstract])) OR (((((((((((eicosapentaenoic acid ethyl ester [Supplementary Concept]) OR (icosapent ethyl[Title/Abstract])) OR (5,8,11,14,17-eicosapentaenoic acid, ethyl ester, (5Z,8Z,11Z,14Z,17Z)-[Title/Abstract])) OR (ethyl all-cis-5,8,11,14,17-icosapentaenoate[Title/Abstract])) OR (ethyl eicosapentaenoate[Title/Abstract])) OR (ethyl icosapentaenoate[Title/Abstract])) OR (ethyl eicosapentaenoic acid[Title/Abstract])) OR (ethyl-EPA[Title/Abstract])) OR (ethyl-eicosapentaenoic acid[Title/Abstract])) OR (Epadel[Title/Abstract])) OR (vascepa[Title/Abstract])) OR (AMR101[Title/Abstract]) |
| 30 | ((bile acid sequestrant*[Title/Abstract]) OR (bile-acid sequestrant[Title/Abstract])) OR (Bile acid sequestrant antilipemic agent[Title/Abstract]) |
| 31 | (((((((((((((((((((((Colesevelam Hydrochloride[MeSH Terms]) OR (Hydrochloride, Colesevelam[Title/Abstract])) OR (Colesevelam HCl[Title/Abstract])) OR (HCl, Colesevelam[Title/Abstract])) OR (Colesevelam[Title/Abstract])) OR ("GT 31104"[Title/Abstract])) OR (31104, GT[Title/Abstract])) OR (GT31-104[Title/Abstract])) OR (GT31104[Title/Abstract])) OR (GT31 104[Title/Abstract])) OR (GT-31104[Title/Abstract])) OR (CholestaGel[Title/Abstract])) OR (Welchol[Title/Abstract])) OR (Cholestyramine Resin*[MeSH Terms])) OR (Resin*, Cholestyramine[Title/Abstract])) OR (Cholestyramine*[Title/Abstract])) OR (Colestyramin*[Title/Abstract])) OR (Cuemid*[Title/Abstract])) OR (Quantalan*[Title/Abstract])) OR (Questran*[Title/Abstract])) OR (MK-135[Title/Abstract])) OR ("MK 135"[Title/Abstract]) |
| 32 | (ATP citrate lyase inhibitor[Title/Abstract]) OR (ACL inhibitor[Title/Abstract]) |
| 33 | ((((((((8-hydroxy-2,2,14,14-tetramethylpentadecanedioic acid [Supplementary Concept]) OR (bempedoic acid[Title/Abstract])) OR (ETC-1002[Title/Abstract])) OR (ESP-55016[Title/Abstract])) OR (nexletol[Title/Abstract])) OR (nilemdo[Title/Abstract])) OR ("ESP 55016"[Title/Abstract])) OR (ETC1002[Title/Abstract])) OR ("ETC 1002"[Title/Abstract]) |
| 34 | (((ALN-PCS [Supplementary Concept]) OR (ALN-PCSsc[Title/Abstract])) OR (inclisiran[Title/Abstract])) OR (leqvio[Title/Abstract]) |
| 35 | #8 OR #9 OR #17 OR #18 OR #19 OR #20 OR #21 OR #26 OR #27 OR #28 OR #29 OR #30 OR #31 OR #32 OR #33 OR #34 |
| 36 | #3 AND #7 AND #35 |
